# Longitudinal Accumulation of Cerebral Microhemorrhages in Dominantly Inherited Alzheimer Disease

**DOI:** 10.1212/WNL.0000000000011542

**Published:** 2021-03-23

**Authors:** Nelly Joseph-Mathurin, Guoqiao Wang, Kejal Kantarci, Clifford R. Jack, Eric McDade, Jason Hassenstab, Tyler M. Blazey, Brian A. Gordon, Yi Su, Gengsheng Chen, Parinaz Massoumzadeh, Russ C. Hornbeck, Ricardo F. Allegri, Beau M. Ances, Sarah B. Berman, Adam M. Brickman, William S. Brooks, David M. Cash, Jasmeer P. Chhatwal, Helena C. Chui, Stephen Correia, Carlos Cruchaga, Martin R. Farlow, Nick C. Fox, Michael Fulham, Bernardino Ghetti, Neill R. Graff-Radford, Keith A. Johnson, Celeste M. Karch, Christoph Laske, Athene K.W. Lee, Johannes Levin, Colin L. Masters, James M. Noble, Antoinette O'Connor, Richard J. Perrin, Gregory M. Preboske, John M. Ringman, Christopher C. Rowe, Stephen Salloway, Andrew J. Saykin, Peter R. Schofield, Hiroyuki Shimada, Mikio Shoji, Kazushi Suzuki, Victor L. Villemagne, Chengjie Xiong, Igor Yakushev, John C. Morris, Randall J. Bateman, Tammie L.S. Benzinger, on behalf of the Dominantly Inherited Alzheimer Network

**Affiliations:** From the Departments of Radiology (N.J.-M., T.M.B., B.A.G., G.C., P.M., R.C.H., T.L.S.B.), Neurology (E.M., J.H., B.M.A., R.J.P., J.C.M., R.J.B.), Psychological and Brain Sciences (J.H.), Psychiatry (C.C., C.M.K.), and Pathology and Immunology (R.J.P.) and Division of Biostatistics (G.W., C.X.), Washington University School of Medicine, St. Louis, MO; Banner Alzheimers Institute (Y.S.), Phoenix, AZ; Department of Cognitive Neurology and Neuropsychology (R.F.A.), Instituto de Investigaciones Neurológicas Fleni, Buenos Aires, Argentina; Departments of Neurology and Clinical and Translational Science (S.B.B.), University of Pittsburgh School of Medicine, PA; Department of Neurology (A.M.B.), Taub Institute for Research on Alzheimers Disease and the Aging Brain, College of Physicians and Surgeons, Columbia University, New York, NY; Neuroscience Research Australia (W.S.B., P.R.S.); School of Medical Sciences (P.R.S.), University of New South Wales (W.S.B.), Sydney, Australia; Dementia Research Centre and UK Dementia Research Institute (D.M.C., N.C.F., A.O.), UCL Queen Square Institute of Neurology, London, UK; Departments of Neurology (J.P.C., K.A.J.) and Radiology (K.A.J.), Massachusetts General Hospital, Boston; Department of Neurology (H.C.C., J.M.R.), Keck School of Medicine of USC, Los Angeles, CA; Department of Psychiatry and Human Behavior (S.C., A.K.W.L., S.S.), Memory and Aging Program, Butler Hospital, Brown University Alpert Medical School, Providence, RI; Center for Neuroimaging, Department of Radiology and Imaging Science (M.R.F., A.J.S.), Department of Pathology and Laboratory Medicine (B.G.), and Indiana Alzheimers Disease Research Center (A.J.S.), Indiana University School of Medicine, Indianapolis; Departments of Molecular Imaging and Neurology (M.F.), Royal Prince Alfred Hospital, University of Sydney, Australia; Department of Neurology (N.R.G.-R.), Mayo Clinic, Jacksonville, FL; German Center for Neurodegenerative Diseases (DZNE) (C.L., J.L., I.Y.); Section for Dementia Research, Hertie Institute for Clinical Brain Research and Department of Psychiatry and Psychotherapy (C.L.), University of Tübingen; Department of Neurology (J.L., I.Y.), Ludwig-Maximilians-Universität München; Munich Cluster for Systems Neurology (SyNergy) (J.L., I.Y.), Germany; Florey Institute and The University of Melbourne (C.L.M.), Australia; Department of Neurology (J.M.N.), Columbia University Irving Medical Center, New York, NY; Department of Radiology (K.K., C.R.J., G.M.P.), Mayo Clinic, Rochester, MN; Department of Molecular Imaging and Therapy (C.C.R., V.L.V.), Austin Health, University of Melbourne, Heidelberg, Australia; Clinical Research Center for Dementia (H.S.), Osaka City University; Department of Neurology (M.S.), Hirosaki University Graduate School of Medicine; and Department of Neurology (K.S.), The University of Tokyo, Japan.

## Abstract

**Objective:**

To investigate the inherent clinical risks associated with the presence of cerebral microhemorrhages (CMHs) or cerebral microbleeds and characterize individuals at high risk for developing hemorrhagic amyloid-related imaging abnormality (ARIA-H), we longitudinally evaluated families with dominantly inherited Alzheimer disease (DIAD).

**Methods:**

Mutation carriers (n = 310) and noncarriers (n = 201) underwent neuroimaging, including gradient echo MRI sequences to detect CMHs, and neuropsychological and clinical assessments. Cross-sectional and longitudinal analyses evaluated relationships between CMHs and neuroimaging and clinical markers of disease.

**Results:**

Three percent of noncarriers and 8% of carriers developed CMHs primarily located in lobar areas. Carriers with CMHs were older, had higher diastolic blood pressure and Hachinski ischemic scores, and more clinical, cognitive, and motor impairments than those without CMHs. *APOE* ε4 status was not associated with the prevalence or incidence of CMHs. Prevalent or incident CMHs predicted faster change in Clinical Dementia Rating although not composite cognitive measure, cortical thickness, hippocampal volume, or white matter lesions. Critically, the presence of 2 or more CMHs was associated with a significant risk for development of additional CMHs over time (8.95 ± 10.04 per year).

**Conclusion:**

Our study highlights factors associated with the development of CMHs in individuals with DIAD. CMHs are a part of the underlying disease process in DIAD and are significantly associated with dementia. This highlights that in participants in treatment trials exposed to drugs, which carry the risk of ARIA-H as a complication, it may be challenging to separate natural incidence of CMHs from drug-related CMHs.

Cerebral microhemorrhages (CMHs) or microbleeds, small bleeds in the brain, are commonly observed in older adults, particularly in those with dementia.^1^ These lesions are commonly related to hypertension,^2^ vascular β-amyloid (Aβ) related to Alzheimer disease (AD) or cerebral amyloid angiopathy (CAA),^3^ or Aβ-modifying therapies currently in AD clinical trials^4^ as part of the constellation of hemorrhagic amyloid-related imaging abnormalities (ARIA-H). The presence of CMHs at baseline predicts future additional CMHs in both the general population^3^ and in AD.^5^ Therefore, during trials of Aβ-modifying therapies, the Food and Drug Administration recommends monitoring for CMHs and excluding participants with 5 or more CMHs.^6^

Because older participants often have mixed pathologies, it can be difficult to discriminate or identify which of these 3 factors is responsible for CMHs, particularly in treatment trials. Evaluating a young cohort, such as individuals with dominantly inherited AD (DIAD),^7,8^ with relatively normal blood pressure and not on treatment may help characterize the natural history of CMHs uniquely related to AD. This may provide a better understanding of the implications of CMHs when observed in individuals with DIAD in treatment trials as well as potentially help evaluate other AD cohorts.

Here, we report results from the Dominantly Inherited Alzheimer Network (DIAN) observational study,^9^ in which participants are assessed for ARIA-H and followed longitudinally with the aim of defining the clinical risks related to CMHs.

## Methods

### Participants

We evaluated 511 participants from 19 DIAN sites using standardized clinical, neuropsychological, and imaging assessments according to DIAN study protocols.^9^ Of the 534 participants with data that passed strict quality control procedures as part of the 14th DIAN Data Freeze (January 2009–December 2019), we selected data based on the availability of MRI and radiology reads from Mayo Clinic. *APP*, *PSEN1*, and *PSEN2* mutation carriers (n = 310, including 109 symptomatic) and noncarriers (n = 201) underwent clinical assessments, neuropsychological testing, and neuroimaging. A subset of 218 carriers, including 76 symptomatic and 127 noncarriers, had at least one follow-up visit and were evaluated longitudinally. These participants had similar clinical characteristics as those without follow-up.

### Standard Protocol Approvals, Registrations, and Patient Consents

Each DIAN site received approval from its institutional review board and its institutional or regional ethical standards committee on human experimentation. All participants or their caregivers provided written informed consent approved by their local institutional review board.

### Clinical Assessment

The protocol used for the clinical assessment is as previously described.^10^ Clinical Dementia Rating (CDR) evaluated the presence (CDR >0, symptomatic) or absence (CDR 0, asymptomatic) of dementia.^11^ The CDR Sum of Boxes (CDR-SB) is a total score of all subcategories of the CDR assessment and was used as a continuous measure.^11^ The estimated years to symptom onset (EYO) was defined as the participant's age at each assessment minus the estimated age at symptom onset for their specific mutation.^7^ Other clinical and vascular-related measures were assessed, including diastolic and systolic blood pressure and mean arterial pressure (MAP). Conditions of hypertension, stroke, hypercholesterolemia, and diabetes were clinically diagnosed. The Hachinski Ischemic Score,^12^ taking into account any history of hypertension and history of stroke, was also assessed. Motor impairment, including assessment of gait and tremor, were also evaluated. An abnormal gait was reported when the participant was unsteady, shuffled, had little or no arm swing, dragged a foot, or had a change in gait not due to injury or arthritis. Clinicians who performed the assessments were not explicitly aware of the mutation status of participants.

### Neuropsychological Testing

Participants underwent a comprehensive battery of neuropsychological tests at each visit, as described previously.^13^ The battery included measures of global cognition, episodic memory, executive function, attention, processing speed, semantic memory, and language. To minimize the number of statistical comparisons, a cognitive composite measure similar to the one used as an endpoint in the DIAN Trials Unit^14^ and in the A4 trial^15^ was included in the analyses. The cognitive composite consisted of the Mini-Mental State Examination global score, Digit–Symbol Substitution from the Wechsler Adult Intelligence Scale–Revised, Logical Memory delayed recall from the Wechsler Memory Scale–Revised, and the delayed recall of the DIAN Word List Test. Scores from each test were transformed to *z* scores using the mean and SD of a relatively healthy cohort that is at least 15 years before estimated age at symptom onset and then averaged to form the cognitive composite score.^16^

### Image Acquisition Protocol

Standardized procedures and protocols, concordant with the Alzheimer Disease Neuroimaging Initiative group, were used at the different DIAN sites to ensure consistency in data collection (adni-info.org). The scanner parameters and image quality were reviewed by the Mayo Clinic in Rochester as previously described.^17^ During baseline and follow-up MRI sessions, all participants underwent on 3T scanners a magnetization-prepared rapid acquisition with gradient echo (resolution 1.0 × 1.0 × 1.2 mm^3^, repetition time [TR] 2,300 ms, echo time [TE] 2.95 ms), a fluid-attenuated inversion recovery (FLAIR) (resolution 0.86 × 0.86 × 5.0 mm^3^, TR 9,000 ms, TE 90 ms), and a gradient recalled echo (GRE)–based sequence, either a T2*-GRE (resolution 0.8 × 0.8 × 4 mm^3^, TR 650 ms, TE 20 ms) or a susceptibility-weighted image (SWI) (resolution 0.7 × 0.7 × 2 mm^3^, TR 28 ms, TE 20 ms). At baseline visit, 240 participants had T2*-GRE and 234 had SWI sequences. Longitudinal scans were coregistered and reviewed concurrently. Due to harmonization among sites and vendors, some participants switched sequence at follow-up from SWI to T2*-GRE (n = 153). The T2*-GRE was tailored to be sensitive to small CMHs with an acquisition time of ∼2.5 minutes.

### Image Processing and Analyses

CMHs, macrohemorrhages, and superficial siderosis were identified on either T2*-GRE or SWI and confirmed by coauthors K.K. and C.R.J. using the same methodology for cross-sectional and longitudinal evaluation previously described by Kantarci et al.^18^ Lesions ≤10 mm that were dissociable from small vessels were counted as definite CMHs. Macrohemorrhages were larger hemorrhagic lesions visible on multiple slices and superficial siderosis was defined by visible signal abnormalities along the pia. The CMH location was reported as deep (basal ganglia, brainstem, thalamus), lobar (supratentorial cortico-subcortical areas of the frontal, parietal, temporal, and occipital lobes), or cerebellar. All findings reported from visual inspection of the images passed quality control from the Mayo Clinic. White matter hyperintensity (WMH) volumes were extracted from FLAIR images using the lesion segmentation toolbox in SPM8.^19^ The cortical thickness of precuneus and inferior parietal, regions known to be highly affected in DIAD,^20,21^ and the hippocampal volume were measured using FreeSurfer software (surfer.nmr.mgh.harvard.edu/).

### Statistical Analysis

Demographic, clinical, and vascular-related measures were compared among noncarriers, carriers without CMHs, and carriers with CMHs for the cross-sectional cohort and among noncarriers, carriers without change in CMH counts, and carriers with increase in CMH counts at follow-up for the longitudinal subset. The mutation carrier group with CMHs was older and more advanced in expected disease stage (analysis of variance and Tukey honestly significant difference [HSD] post hoc tests). Thus, age and family age at onset were included as covariates in analysis of covariance/Tukey HSD and logistic regression/Wald χ^2^ tests for group comparisons of continuous and dichotomous variables, respectively.

Cross-sectional analyses evaluated the prevalence and location of CMHs in carriers and noncarriers. Multivariable logistic regression analyses were used to evaluate factors associated with the prevalence of CMHs (dichotomized as yes vs no) in noncarriers and carriers with the stepwise selection method. The goodness of model fit was evaluated using the Hosmer and Lemeshow test.^22^ Because of the relatively small number of events, the penalized maximum likelihood method was used to estimate the parameters and profile likelihood confidence intervals (CIs) were reported for odds ratios (ORs).^22^ Fisher exact tests investigated effect of mutation types (*PSEN1*/*PSEN2*/*APP*) and subtypes (*PSEN1* mutation before/after codon 200 and *APP*-Dutch/*APP*-non-Dutch) on prevalent or incident CMHs.

To determine whether the number of CMHs at baseline influenced the rate of incident CMHs in carriers, the severity of CMH finding was categorized as follows: no (0 CMHs), mild (1 CMH), moderate (2–4 CMHs), and severe (more than 4 CMHs). Similar categorization was used previously to evaluate association of CMHs with cognitive decline.^23^ Generalized linear mixed effects (LME) models were used to estimate the rate of change in CMHs for asymptomatic carriers and symptomatic carriers and to evaluate whether the presence of CMHs at baseline (dichotomized as yes vs no), its increase (dichotomized as yes vs no), or the number of CMHs at baseline, respectively, were associated with the longitudinal change in cortical thickness, hippocampal volume, WMH volumes, CDR-SB, and the cognitive composite among carriers.^24^ To discount potential effects of pathology in Dutch mutation carriers, all analyses were also run excluding this group. The type of scanner sequence (T2*-GRE, SWI, and SWI to T2*-GRE) was controlled for in the longitudinal analyses. Sensitivity analyses were also performed to investigate the effect of other factors such as baseline age, baseline EYO, *APOE* ε4 status, sex, MAP, and sequence type. The normality assumption was examined using histograms and the Q-Q (quantile-quantile) plots of the residuals output by the mixed effects models.

All analyses were conducted using SAS 9.4 (SAS Institute Inc., Cary, NC) or R (r-project.org). Missing data during the longitudinal follow-up were considered missing at random. All tests are 2-sided and *p* values less than 0.05 are considered significant.

### Data Availability

The DIAN data are available upon request. All requests for data must be submitted in writing via the electronic data request form available at dian.wustl.edu.

## Results

### Participant Clinical and Vascular-Related Characteristics in the Presence of CMHs

The demographics, along with clinical, psychometric, and vascular-related variables of the cross-sectional and longitudinal cohorts, are summarized in tables 1 and 2. The clinical assessments based on CDR and CDR-SB did not show differences between carriers with and without CMHs, while cognitive assessment with the cognitive composite measure revealed that carriers with CMHs were more impaired than carriers without CMH and noncarriers (table 1). Carriers with an increase in CMH at follow-up were also more cognitively impaired compared to carriers without change in CMH based on the cognitive composite. These 2 groups were similar in clinical measurements (table 2). It is important to note that carriers with CMHs and with increased CMH at follow-up were significantly older and at a more advanced EYO (tables 1 and 2). After controlling for age, carriers with CMHs had higher prevalence of abnormal gait (table 1), but the prevalence of tremor, diabetes, and hypercholesterolemia was similar in the 3 groups (table 1). Mean arterial blood pressure, diastolic blood pressure, and Hachinski Ischemic Score were significantly higher in carriers with CMHs (table 1). Carriers with an increase in CMH count at follow-up presented higher mean arterial blood pressure, diastolic and systolic blood pressure, and Hachinski Ischemic score compared to carriers without change in CMH at follow-up (table 2). A stroke history was observed only in carriers with CMHs.

**Table 1:**
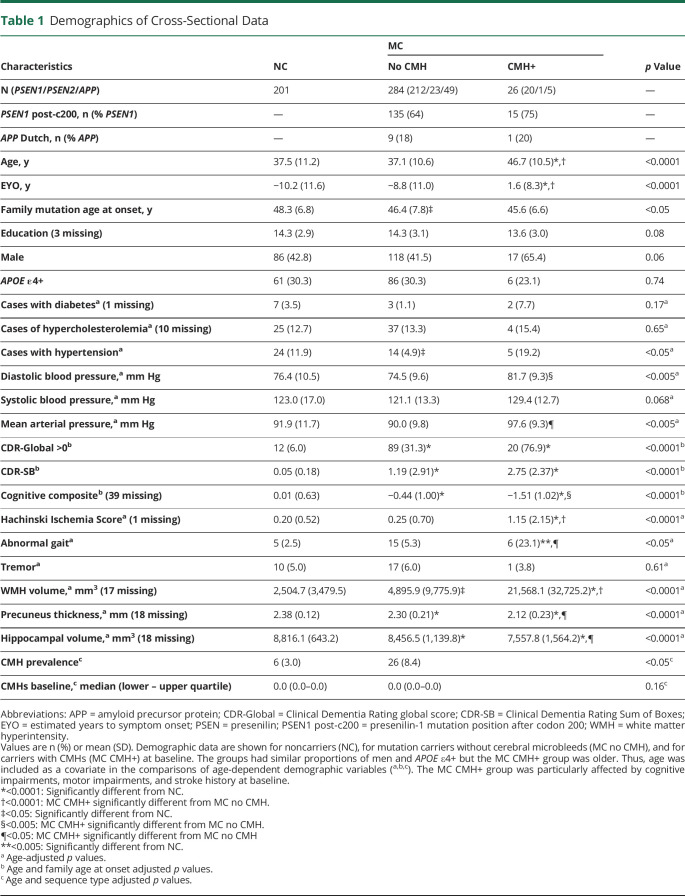
Demographics of Cross-Sectional Data

**Table 2:**
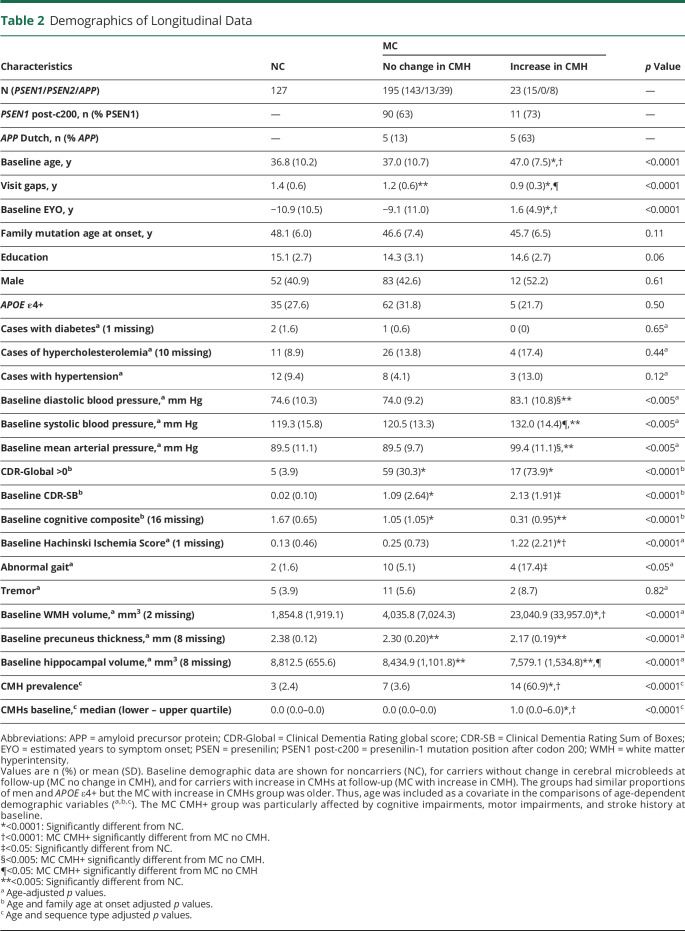
Demographics of Longitudinal Data

### CMH Prevalence and Location

**Figure 1:**
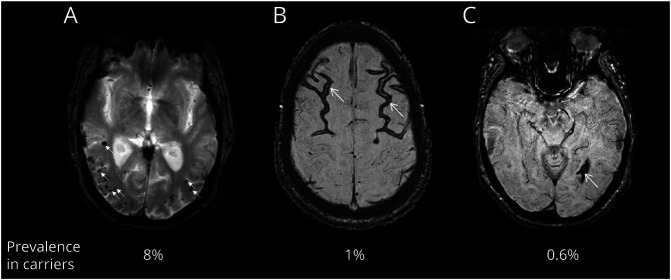
Hemorrhagic Amyloid-Related Imaging Abnormality Observed on Gradient Echo MRI in Mutation Carriers (A) Severe cerebral microhemorrhage (CMH) lesion observed in posterior cortico-subcortical area (arrows indicate several CMHs) on T2* gradient recalled echo. (B) Severe superficial siderosis (arrows) detected on susceptibility-weighted image (SWI). (C) Macrohemorrhage (arrow) observed in temporal area on SWI. In carriers, CMHs (A), superficial siderosis (B), and macrohemorrhages (C) are observed with an overall prevalence of 8%, 1%, and 0.6%, respectively.

Of all 511 participants, 32 (6.3%) participants had CMHs at baseline. Of the 310 carriers, 26 (8.4%) had CMHs, whereas of the 201 noncarriers, only 6 (3.0%) had CMHs. The CMH prevalence was 3.0% and 18.3% in asymptomatic and symptomatic carriers, respectively. Thirteen participants with baseline SWI had 32 CMHs overall; upon protocol change to T2*-GRE, the same 32 CMHs were again detected, along with additional new CMHs. Stepwise selection of multivariable logistic regression analysis indicated that carriers were more likely to have CMHs compared with noncarriers (OR 3.575, 95% CI 1.499, 9.904). Age and diastolic blood pressure were also significantly associated with the odds of having CMHs (OR 1.071, 95% CI 1.034, 1.110 and OR 1.068, 95% CI 1.030, 1.108, respectively). Controlling for these factors, CDR, systolic blood pressure, MAP, sex, *APOE* ε4 status (yes vs no), sequence type, and education were not significantly associated with the odds of having CMHs. Within carriers, the mutation type was not associated with the odds of having CMHs. When looking at CMH severity, a count of 5 or more CMHs was identified in 1.6% of the overall cohort, all of them mutation carriers (n = 8). CMHs were found more in lobar areas than in deep areas, and preferentially in occipital areas (figure 1A). Eighty-one percent of the participants with CMHs (n = 26) had lesions strictly in lobar areas while 3% had CMHs only in deep areas. Sixteen percent had CMHs in both lobar and deep areas. Only 2 participants (0.6%) developed CMHs solely located in the cerebellum.

### CMHs, Other ARIA-H, and White Matter Lesions

Besides CMHs, other types of hemorrhagic lesions were also observed on GRE MRI in carriers. Though present in fewer cases, these lesions can be severe (figure 1). Superficial siderosis, corresponding to subarachnoid hemorrhage, was detected in 4 participants with and without CMHs (figure 1B). The 4 participants were from different mutation types (2 *PSEN1* and 2 *APP* mutation carriers); 3 were symptomatic and had or developed new CMHs. The most severe case demonstrated widely spread superficial siderosis in the occipital lobe; this participant was an *APP* mutation carrier with severe CMH counts (31, including 10 in the occipital) and a macrohemorrhage in the occipital lobe. Macrohemorrhages were overall observed in 2 *APP* mutation carriers, both with CMHs and history of stroke (figure 1C). The other *APP* carrier with macrohemorrhages was a Dutch mutation carrier and presented with moderate CMH severity at baseline. Interestingly, the participants with the highest number of CMHs in the overall cohort (135 CMHs at baseline) and the highest increase in CMHs (3–139 CMHs over 7 years) did not have any superficial siderosis or macrohemorrhages, suggesting that the severity of CMHs is not always a predictor of the presence of those abnormalities. However, concerning white matter lesions (quantified here as WMH volume), we observed that the severity of CMH in mutation carriers was associated with increased white matter lesion volume (*p* < 0.0001, *F*_*3,208*_ = 26.2) and with larger volume increase at follow-up (*p* = 0.001, *F*_*3,244*_ = 5.4), particularly when 5 or more CMHs were present at baseline (14,847.1 mm^3^/y, *p* < 0.0001, 95% CI 7,555.6, 22,138.6). Similar results were observed when Dutch mutation carriers were excluded.

### CMH Increase and Baseline Status

**Figure 2:**
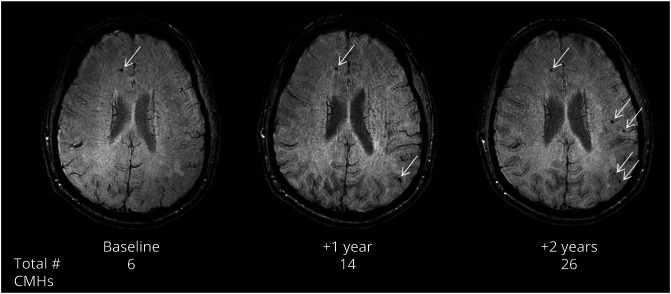
Longitudinal Accumulation of Cerebral Microhemorrhages (CMHs) in a Mutation Carrier Baseline, 1 year, and 2 year follow-up susceptibility-weighted image scans of 1 participant showing longitudinal accumulation of CMHs (arrows). The total number of CMHs observed on the overall scan was 6, 14, and 26, respectively.

A total of 345 participants had longitudinal data with 2 or more visits over 0.9–9.1 years, with an average of 1.3 ± 0.6 years between evaluations. Three (2.4%) of the 127 noncarriers developed new CMHs during the follow-up, whereas 23 (10.6%) of the 218 carriers developed new CMHs (figure 2). Among carriers, 14 (66.7%) of the 21 with CMHs at baseline developed new CMHs during the follow-up, whereas only 9 (4.6%) of the 197 carriers without CMH at baseline developed new CMHs (*p* < 0.0001, Fisher exact test, OR 35.6, 95% CI 12.9, 98.6). Although some mutations presented with +5 CMH/year (table 3), the odds of increased CMHs was not influenced by the mutation type (*PSEN1*, *PSEN2*, or *APP*). Subgroup analyses showed that Dutch mutation carriers have higher odds of developing new CMHs compared to *APP* Dutch noncarriers (*p* < 0.005, Fisher exact test, OR 5.8, 95% CI 1.8, 22.6). No association was found regarding the mutation position within *PSEN1*.

**Table 3:**
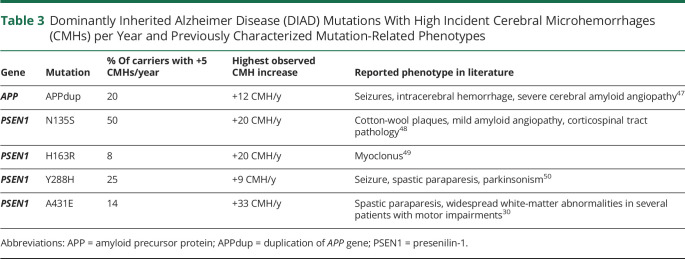
Dominantly Inherited Alzheimer Disease (DIAD) Mutations With High Incident Cerebral Microhemorrhages (CMHs) per Year and Previously Characterized Mutation-Related Phenotypes

**Figure 3:**
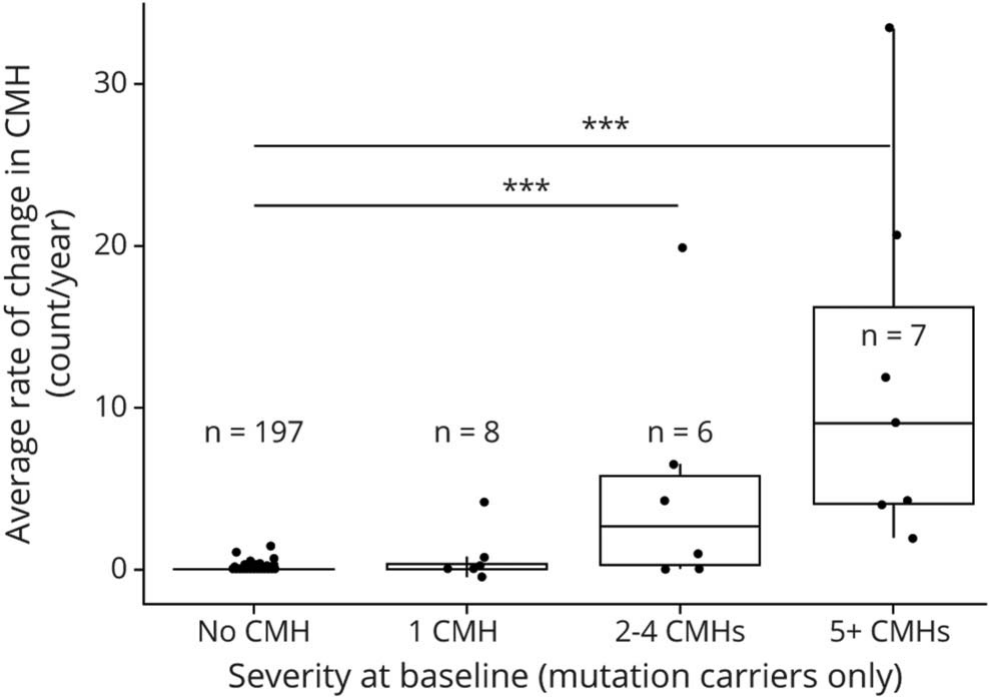
Increase of Cerebral Microhemorrhages (CMHs) as a Function of the Number of CMHs at Baseline in Carriers In mutation carriers (n = 218), the rate of CMH accumulation per year was different as a function of the number of CMHs observed at baseline. The presence of 2–4 CMHs (moderate CMH count) and more than 5 CMHs (severe CMH count) at baseline predicted a higher rate of increase in CMHs at follow-up (5.9 ± 0.9 and 11.2 ± 0.9 CMHs per year, respectively) relative to the group with no CMH at baseline. ***p < 0.0001.

For all participants with CMHs at baseline, the rate of increase in CMHs per year was 0.01 ± 0.04 for noncarriers and 0.58 ± 3.18 for carriers overall, but 8.95 ± 10.04 for the 13 carriers with 2 CMHs or more at baseline. In carriers, the rate of CMH accumulation per year was associated with the number of CMHs observed at baseline (*p* < 0.0001, *F*_*3,252*_ = 57.5). The presence of 2–4 CMHs and more than 4 CMHs at baseline were associated with higher rate of increase per year (5.9 CMH/year, *p* < 0.0001, 95% CI 4.0, 7.7 and 11.2 CMH/year, *p* < 0.0001, 95% CI 9.4, 13.1 CMHs, respectively) (figure 3). Similar associations were observed when excluding Dutch mutation carriers.

**Figure 4:**
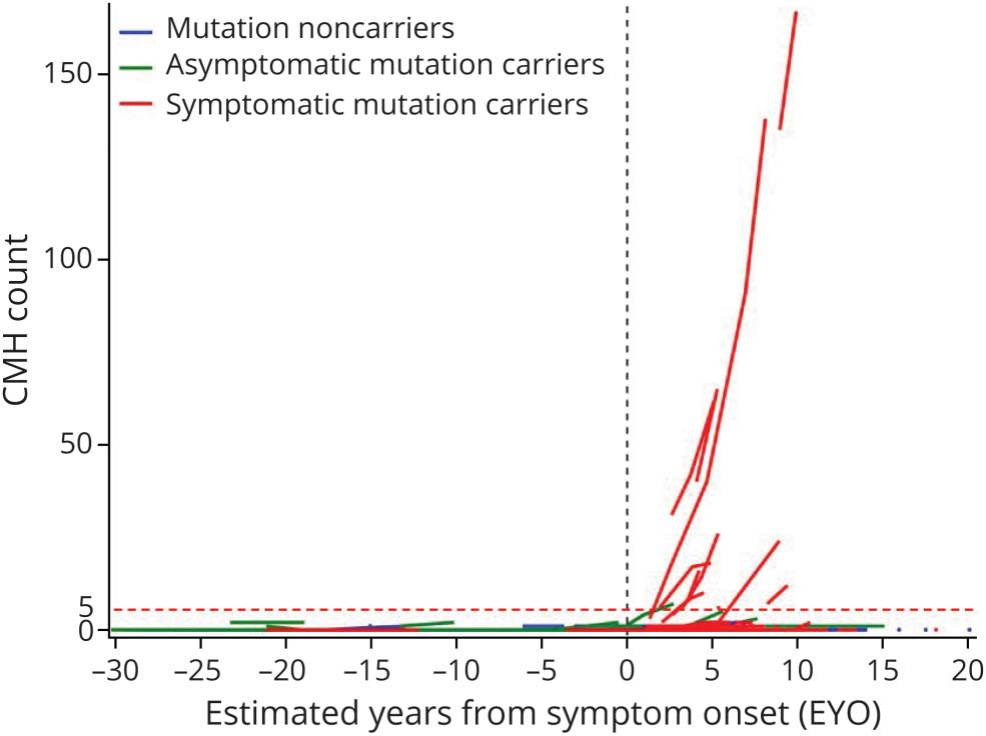
Longitudinal Accumulation of Cerebral Microhemorrhages (CMHs) as a Function of Estimated Years to Symptom Onset Plot of CMH count as a function of estimated years from symptom onset (EYO) within mutation noncarriers (blue, n = 127), asymptomatic mutation carriers (green, n = 142), and symptomatic mutation carriers (red, n = 76). Accumulation of CMH is more pronounced (increase to >5 CMHs, above red dashed line) and mostly observed in symptomatic mutation carriers (n = 17, 22.4%) past EYO 0 (black dashed line). Three noncarriers (2.4%) and 6 asymptomatic mutation carriers (4.2%) had new CMHs to a lesser extent (increase to <5 CMHs, under red dashed line).

Figure 4 shows an individual's longitudinal CMH count as a function of EYO. All participants with more than 2 CMHs and with an increased rate of 2 or more CMHs per year were symptomatic carriers (figure 4). For carriers, the rate of CMH occurrence increased near the expected date of symptom onset (figure 4). Based on the generalized linear mixed effects model analysis, only the symptomatic carriers had an increased rate in CMHs during the follow-up (1.62 CMH/year, *p* < 0.0001, 95% CI 0.95, 2.29). Similar analyses for *APOE* ε4 status revealed that *APOE* ε4 status (noncarrier vs carrier) does not influence the annual CMH increase whether accounting for Dutch mutation carriers or not. Controlling for clinical symptomatic status, sensitivity analyses indicated that baseline age, baseline MAP, change of sequence type during the follow-up, *APOE* ε4, and sex were not associated with the change in CMHs.

### CMH Presence/Increase and Disease Progression

Among the carriers with longitudinal follow-up, participants with CMHs or with an increase in CMHs at follow-up were more advanced in expected disease stage. Adjusting for baseline EYO, the LME model showed that having CMHs at baseline or having an increase in CMHs at follow-up was not associated with faster cortical thinning of precuneus or inferior parietal, hippocampal volume decrease or faster change in white matter lesion volumes. Moreover, participants who had CMHs at baseline demonstrated faster increase in CDR-SB (0.67/y, *p* = 0.001, 95% CI 0.27, 1.07) and a larger, but nonsignificant, annual decline in the cognitive composite (−0.07/y, *p* = 0.14, 95% CI −0.16, 0.02) compared with those without CMH at baseline. Similarly, carriers with increased CMHs during the follow-up had a faster annual increase in CDR-SB (0.75/y, *p* = 0.048, 95% CI 0.01, 1.49) and a larger, but nonsignificant, annual decline in the cognitive composite (−0.07/y, *p* = 0.38, 95% CI −0.21, 0.08) compared to those without an increase in CMHs. Analyses excluding Dutch mutation carriers did not alter these findings.

## Discussion

In this cross-sectional and longitudinal study, we describe microhemorrhage prevalence, location, severity, and inherent increase in a population with DIAD, allowing study of AD pathology with reduced influence from confounders of age-related vascular risk factors. We found that CMH were associated with worsening of clinical symptoms, occurred primarily after the expected age at symptom onset, and that, once present, they are likely to increase in number over time. These findings have important implications for participant selection and monitoring in clinical trials involving the DIAD population.

Hypertension is a risk factor for cerebral hemorrhagic lesions and a common feature of AD.^2^ Carriers with CMHs had mildly elevated blood pressure (MAP 97.6 ± 9.3 mm Hg) and the presence of CMHs was associated with higher diastolic and mean arterial pressure. However, blood pressure values were not associated with accumulation of CMHs or cognitive decline. These findings suggest that higher blood pressure may still contribute to CMHs in a relatively young population with a significant risk for AD. CMHs were mainly located in lobar areas with a minority of deep CMHs, supporting the hypothesis that CMHs in the setting of DIAD are largely due to CAA rather than hypertensive arteriopathy.^25^ A recent study from Graff-Radford et al.^26^ showed that Aβ burden is associated with lobar CMHs but not deep CMHs in aging populations, which supports CAA as the pathologic substrate for multiple lobar CMHs. Previous studies showed predominance for lobar CMHs in familial and sporadic AD populations.^1,27^ CAA has been observed in DIAD populations^28^ with higher frequency than in sporadic AD,^29^ but further postmortem evaluation is needed to correlate CAA and CMHs in DIAD. Diabetes and hypercholesterolemia were not found more frequently in carriers with CMHs.

Regarding clinical risks, carriers with CMHs in our study had more severe gait disorders and cognitive impairment at baseline. CMHs were previously reported in carriers of specific DIAD mutations associated with parkinsonism and spastic paraparesis.^30^ It has also been reported that the presence of lobar CMHs predicted worsening of gait and parkinsonism in patients with dementia, including AD.^1^ In our DIAD cohort, abnormal gait but not tremor was associated with the presence of CMHs. Further study is of interest to better establish the relationship between motor deterioration and CMH in DIAD. Symptomatic carriers were most likely to develop CMHs and were the only group with a significant increase in CMHs per year. Only a few occurrences of CMHs were observed before the estimated year of symptoms onset, suggesting a relationship between disease progression and occurrence of CMHs. A large study involving 3,257 participants showed that the presence of CMHs was associated with an increased risk for dementia, including AD, and that the presence of more than 4 CMHs was associated with cognitive decline.^23^ In our study, DIAD mutation carriers declined faster as measured by CDR-SB when they had CMHs or developed more CMHs.

Our cross-sectional cohort was larger than the longitudinal one due to recent participants having only baseline evaluations and due to participants' transfer from the DIAN Observational Study to the DIAN Trials Unit. However, the 2 cohorts presented similar clinical characteristics, suggesting comparability between cross-sectional and longitudinal analyses. As an international, multisite, longitudinal study, we harmonize protocols across sites. In our dataset, participants had either SWI or T2*-GRE sequences at each evaluation, with some sites changing from SWI to T2*-GRE for harmonization. Although the T2*-GRE employed was specifically tailored for sensitivity (∼2.5 minutes acquisition time instead of traditional 30-second scan), this raises concerns regarding differences in detection sensitivity.^31^ All CMHs observed on SWI were detected on subsequent T2*-GRE, suggesting no underdiagnosis of CMHs with the change in protocol. Moreover, sensitive analyses showed no effect of sequence type on CMH detection.

The prevalence of CMHs varies across studies on DIAD populations. Whereas Ryan et al.,^27^ in a study including 12 individuals with DIAD, observed 25% prevalence of CMHs, a previous DIAN study of 175 participants reported a 15% prevalence of CMHs.^32^ Here we evaluated 511 participants and found around 8% of mutation carriers with CMHs. Note that the mean age of these studies on DIAD is below 50 years while studies on sporadic AD reported a prevalence of CMHs of 23%–24% in cohorts with a mean age above 67 years.^33^

In our study, age was an important factor for CMH prevalence even though the group is relatively young (46.7 ± 10.5 years for carriers with CMHs). Thus, the age of our population and the variability in CMHs counts raise the question of the role of genetic factors.^34^ Several studies reported increased risk of vascular disease associated with specific mutations, particularly within the *APP* and *PSEN1* genes.^27^ The *APP* Dutch mutation (Glu693Gln) is associated with CAA and cerebral hemorrhage, reporting CMH prevalence of more than 60%.^35^ *PSEN1* mutations after the codon 200 are possibly associated with severe CAA.^36^ Using Exact tests for our small group size, the odds of having CMHs or developing new CMH was not influenced by mutation type (*PSEN1*, *PSEN2*, or *APP*) among carriers. Analyses of subgroups within *APP* (Dutch vs non-Dutch) or *PSEN1* (before vs after codon 200) mutation carriers revealed increased risk for incident CMHs in Dutch mutation carriers. However, none of the findings excluding Dutch mutation carriers were different, suggesting that our findings were not driven by this group. The *APOE* ε4 allele has been shown to be associated with increased risk for developing CMHs^37,38^ but this risk was not necessarily found in relation to the Dutch mutation.^39^ In our study, though we could not establish a clear relationship with specific mutation types, the *APOE* ε4 allele was not associated with increased risk for developing CMHs regardless of the inclusion of Dutch mutation carriers. These findings demonstrate the variability of genotype–phenotype relationships and the difficulty of relying on genotype to estimate risks for vascular disorders in disease progression and during clinical trials.

Previous studies showed that the presence of CMHs was associated with the presence of superficial siderosis^40^ and indicates increased risk for future macroscopic hemorrhages.^41,42^ A study in normal aging reported a 0.2% prevalence of superficial siderosis with 23% of cases with superficial siderosis also having CMHs.^43^ In our study, 0.19% of the overall cohort had superficial siderosis and half of those with widely spread lesions had CMHs. The 2 participants in the current study with macrohemorrhages had a history of stroke and CMHs with differing severity (2 and 31 lobar CMHs). However, participants with the highest CMH counts (135 lobar CMHs) and with the greatest rate of change in CMHs did not have any superficial siderosis, macrohemorrhages, or history of stroke, suggesting that a high number of CMHs is not necessarily associated with preexisting superficial siderosis or macrohemorrhages.

Whereas in this study we focused on ARIA-H, previous studies found that ARIA edema types (ARIA-E) are closely associated with the presence of CMHs. In our cohort, only one participant had possible ARIA-E and we found no clear evidence for a relationship with CMHs. WMH also observed on FLAIR is a small vessel disease–related abnormality more commonly observed in AD,^44^ including DIAD.^45^ A previous DIAN study demonstrated that white matter lesions were greater in individuals with CMHs compared to those without CMHs and the presence of CMHs was associated with increased WMH volume.^32^ We confirmed this finding with our dataset. Having 5 or more CMHs was particularly associated with change in WMH volume. However, having incident CMHs was not associated with worsening WMH.

Mutation carriers were particularly at risk for developing CMHs and the presence of CMHs at baseline was related to risk for increase in CMH prevalence within 2 years. The presence of CMHs is itself a risk for an increase in CMHs without any treatments, as shown by the accumulation of CMHs over time in our population followed longitudinally. Studies in a healthy elderly population,^3^ preclinical AD,^38^ and a memory clinic population^46^ showed that occurrence of new CMHs is linked to baseline CMHs and reported ORs from 5 to 8, suggesting predictable development of new CMHs when CMHs are present at baseline. Here, we report a smaller OR of 3.6 for our cohort. However, while those studies reported that 10%–12% of participants developed new CMHs, we observed that 66.7% of the carriers who already had CMHs developed new CMHs at follow-up in our DIAD population. Incidence of CMHs was not associated with progressive brain atrophy but was associated with worsening dementia as measured by CDR-SB. The exponential and quasisystematic increase of CMHs makes these findings critical for clinical trial monitoring. For participants in clinical trials at risk for developing ARIA-H as a complication, it will be important to try to separate the natural incidence of CMHs from adverse events related to therapy. Aβ-modifying therapies, such as passive immunotherapy with bapineuzumab, induced CMHs in patients with AD and studies confirmed that the odds to develop CMHs after treatment were increased.^37^ Based on these clinical trial outcomes, AD working groups recommended excluding participants with more than 5 CMHs (defined in our study as a severe CMH finding).^6^ In DIAD, having 2–4 CMHs (defined as moderate CMH severity) is a risk factor for developing more and the odds of increase appear higher than what has been reported in sporadic AD. Based on these results, recommendation guidelines for CMHs in such populations may need to be revisited and adapted for familial AD. This study additionally shows that any clinical trial on individuals with DIAD needs careful participant selection and monitoring.

## Glossary

Aβ: β-amyloid
AD: Alzheimer disease
ARIA-E: edema type amyloid-related imaging abnormalities
ARIA-H: hemorrhagic amyloid-related imaging abnormalities
CAA: cerebral amyloid angiopathy
CDR: Clinical Dementia Rating
CDR-SB: Clinical Dementia Rating Sum of Boxes
CI: confidence interval
CMH: cerebral microhemorrhage
DIAD: dominantly inherited Alzheimer disease
DIAN: Dominantly Inherited Alzheimer Network
EYO: estimated years to symptom onset
FLAIR: fluid-attenuated inversion recovery
GRE: gradient recalled echo
HSD: honestly significant difference
LME: linear mixed effects
MAP: mean arterial pressure
OR: odds ratio
SWI: susceptibility-weighted image
TE: echo time
TR: repetition time
WMH: white matter hyperintensity
